# Computational Simulation of Equivalence Class Formation Using the go/no-go Procedure with Compound Stimuli

**DOI:** 10.1007/s40732-016-0184-1

**Published:** 2016-05-19

**Authors:** Renato Roberto Vernucio, Paula Debert

**Affiliations:** Instituto de Psicologia, Universidade de São Paulo, São Paulo, Brazil

**Keywords:** Simulation, Artificial intelligence, Equivalence, Compound stimulus, Go/no-go procedure

## Abstract

Research about equivalence has commonly utilized human participants as experimental subjects. More recently, computational models have been capable of reproducing performances observed in experiments with humans. The computational model often utilized is called RELNET, and it simulates training and testing trials of conditional relations using the matching-to-sample procedure (MTS). The differentiation between sample stimulus and comparison stimuli, indispensable in MTS, implies operational difficulties for simulations. For this reason, new studies seek to utilize alternative procedures to MTS, which do not differentiate the functions of the antecedent stimuli. This work evaluated the possibility of developing a new computational model to simulate equivalence class formation using the go/no-go procedure with compound stimuli. In Experiment 1, artificial neural networks were utilized to simulate training of the AB and BC relations as well as the testing of the AC relation. The results showed that four out of six runs demonstrated equivalence class formation. Experiment 2 evaluated whether the additional class training performed in Experiment 1, which was analogous to the simulation of pre-experimental experience of human participants, would be essential for simulating the establishment of equivalence classes. It was found that it was not possible to simulate equivalence class formation without the additional class training. Altogether, the experiments show that it is possible to simulate equivalence class formation using the go/no-go procedure with compound stimuli and that it is necessary to conduct additional class training. The model developed is, therefore, an alternative to RELNET for the study of equivalence relations using computational simulations.

Training conditional relations may produce the emergence of new relations that have not been directly trained. Such relations among stimuli possess specific properties denominated reflexivity, symmetry, transitivity and equivalence. When there is evidence that all these relations are established, it is considered that an equivalence class has been formed (Sidman & Tailby, 1982).

Research about equivalence class formation has commonly utilized human participants as experimental subjects (Sidman, 1994). More recently, research has been developed utilizing computational models (artificial neural networks) to simulate equivalence class formation (e.g., Barnes & Hampson, 1993; Cullinan, Barnes, Hampson, & Lyddy, 1994; Lyddy & Barnes-Holmes, 2007; Lyddy, Barnes-Holmes, & Hampson, 2001; Okada, Sakagami, & Yamakawa, 2005; Tovar & Torres, 2012). These models are capable of reproducing performances observed in humans, having the advantage of providing greater control over the experimental variables, creating a stable and controllable environment that could be difficult to obtain in experiments with humans and other animals (Lyddy & Barnes-Holmes, 2007; McClelland, 2009; Tovar & Torres, 2012).

Artificial neural networks are sets of interconnected processing units, generally organized in layers. The set of units that forms the computational representation of the stimuli presented in a particular trial is called the *input layer*, and the set of units that form the representation of the response emission is called the *output layer*. The other layers between them are called *intermediate* or *hidden*. In a feed-forward network architecture, the units of the input layer are connected to the units of the hidden layers, which in turn are connected to the units of the output layer. Between two units there is a weighed connection. When a value passes from a unit to another, it is multiplied by a variable called *connection weight*. The flow of information is unidirectional, from the input to the output layer, in such a way that an input vector is mathematically transformed into an output vector (see Haykin, 2009). A simulation of equivalence class formation, therefore, basically consists of three components: a set of input vectors that represents stimuli presentation, a set of output vectors that represents response emission and a learning algorithm that changes the connection weights so that the input vectors can generate appropriate output vectors.

The computational model often utilized in equivalence research is called RELNET, which simulates training and testing trials using the MTS procedure (e.g., Barnes & Hampson, 1993; Cullinan, Barnes, Hampson, & Lyddy, 1994; Lyddy & Barnes-Holmes, 2007; Lyddy, Barnes-Holmes, & Hampson, 2001). Lyddy and Barnes-Holmes (2007) used computational simulations to comparatively evaluate the performance of equivalence class formation using MTS with linear and one-to-many protocols. Two experimental conditions were simulated. Using the linear protocol, the relations AB and BC were trained, and it was tested whether the relation CA would emerge. For the one-to-many protocol, the relations AB and AC were trained and the emergence of BC and CB tested. As a result, it was found that the simulations that used the linear protocol needed approximately twice the time to finish training. In the testing phase, only the simulations that used the one-to-many protocol showed emergence of the tested relations. Together, these results suggested that the one-to-many protocol is more effective in producing equivalence classes than the linear protocol. A similar conclusion was also found by experiments involving humans (e.g., Arntzen & Holth, 1997).

Training and testing phases in computational simulations are analogous to training and testing phases in experiments with humans, as both have relations established during training and new relations evaluated in the testing phase. However, the procedures are not directly similar. In computational simulations, the training phase consists in using a learning algorithm to make the input vectors generate output vectors progressively closer to those desired. Lyddy and Barnes-Holmes (2007) used the learning algorithm called *backward propagation* (Rumelhart, Hinton, & Williams, 1986). In each iteration, an input vector was passed to the neural network, which was the computational representation of a MTS trial: presentation of a sample and three comparison stimuli. This input vector was mathematically transformed into the output vector, which was the computational representation of choosing one of the comparison stimuli in that trial. In the beginning, the output vectors were not generated according to what was expected. This way, the learning algorithm repeatedly altered the connection weights to produce output vectors closer to those desired. When all input vectors were correctly producing the expected output vectors, the training phase ended and the learning algorithm was disabled, maintaining the connection weight constant. During the testing phase, new input vectors, previously unseen, were passed to the neural network. These new input vectors corresponded to MTS trials that evaluated the emergence of new relations. If these input vectors produced output vectors that revealed established relations that were not directly trained, it was considered that equivalence class formation was simulated.

In RELNET, the differentiation between sample and comparison stimuli occurs in the input layer using a computational resource denominated sample-marking duplicator. Tovar and Torres (2012) pointed out that the way this differentiation is made entails operational problems. In different trials, the units referring to the sample-marking duplicator remain constant and determine the output values. Thus, the results presented in the testing phase are not necessarily determined by the information about the sample and comparison stimuli in each trial, but rather by the information about the sample-marking duplicator. This would decharacterize the network results derived from RELNET as emergent responses. The network’s operational problem derived from RELNET, therefore, arises from the need to differentiate in the input vector which is the sample stimulus and which are the comparison stimuli, a characteristic indispensable in the MTS procedure. For this reason, Tovar and Torres (2012) indicated as a possible solution the development of a new computational model that would simulate equivalence class formation using some alternative procedure to MTS. This new model should not differentiate sample and comparison stimuli. The authors suggested the yes-no and the go/no-go with compound stimuli procedures as possible alternatives to the use of MTS in computational simulations.

Using the yes-no procedure, Tovar and Torres (2012) evaluated the possibility of simulating equivalence class formation without differentiating sample and comparison stimuli functions. In each trial, a set of values was attributed to the input layer units (input vector), which represented the presentation of two stimuli, without specifying their functions or spatial locations. At the end of the trial, the output layer units generated a set of values (output vector) that corresponded to the representation of the *yes* response or the *no* response. To simulate the pre-experimental history of human participants, an additional class training was conducted in which the relations XY, YZ and XZ were established, as performed by Lyddy and Barnes-Holmes (2007). In the training phase, the relations AB and BC for two sets of stimuli were also established using the backward propagation learning algorithm (Rumelhart, Hinton, & Williams, 1986). In the testing phase, the emergence of the AC relation was evaluated. As a result, it was found that five out of six runs demonstrated emergence of the AC transitivity relation and therefore presented equivalence class formation. In order to evaluate the importance of simulating pre-experimental knowledge in this new model, the authors repeated the simulation procedure excluding the steps referring to the additional class training. Upon removal of the additional class training, it was not possible to simulate equivalence class formation.

The model developed by Tovar and Torres (2012) did not need to utilize a similar resource to the sample-marking duplicator, characteristic of the networks derived from RELNET, once it did not differentiate sample and comparison stimuli. The output vectors were generated exclusively by the information about the stimuli present in each trial in such a way that the results obtained in the testing phase represented emergent responses. Therefore, the model proposed by Tovar and Torres (2012) can be considered a valid alternative to RELNET for studies involving simulations of equivalence class formation.

However, the yes-no procedure may generate undesirable effects in experiments with humans because the use of the *yes* and *no* labels can make the participants respond *no* in all the transitivity and equivalence test trials, given that the tested relations were not presented in the training phase (Fields, Reeve, Varelas, Rosen, & Belanich, 1997; Fields, Doran, & Marroquin, 2009). Ideally, it would be desirable for computational simulations to make use of a procedure that, in humans, did not present the type of problem pointed out by Fields et al. (1997) and also did not differentiate sample and comparison stimuli.

Another alternative procedure to MTS, which also does not differentiate sample and comparison stimuli, is the go/no-go procedure with compound stimuli (e.g., Debert, Matos, & McIlvane, 2007; Perez, Campos, & Debert, 2009; Debert, Huziwara, Faggiani, De Mathis & Mcilvane, 2009; Campos, Debert, Barros & McIlvane, 2011; Grisante et al., 2013). The main difference between the procedures yes-no and go/no-go with compound stimuli is the response required by the participant. In the go/no-go procedure with compound stimuli, the participant must emit the response topography previously defined by the experimenter as being the *go* response (e.g., clicking with the mouse, pressing a key, etc.) to compounds with elements from the same class, and the participant must emit the response defined as *no-go*, which would be any other response except the response defined as *go*, to compounds with elements from different classes. Considering that ideally it is desirable to work in computational simulations with procedures that have proven to be successful with humans, it would be advantageous to evaluate the possibility of simulating equivalence class formation with the go/no-go procedure with compound stimuli.

This work aimed to evaluate the possibility of utilizing artificial neural networks to simulate equivalence class formation using the go/no-go procedure with compound stimuli.

## Experiment 1

Experiment 1 evaluated the possibility of utilizing artificial neural networks to simulate equivalence class formation with the go/no-go procedure with compound stimuli. The Tovar and Torres (2012) method was taken as the basis for the development of a new computational model, adapted to the go/no-go procedure with compound stimuli.

### Method

**Fig. 1:**
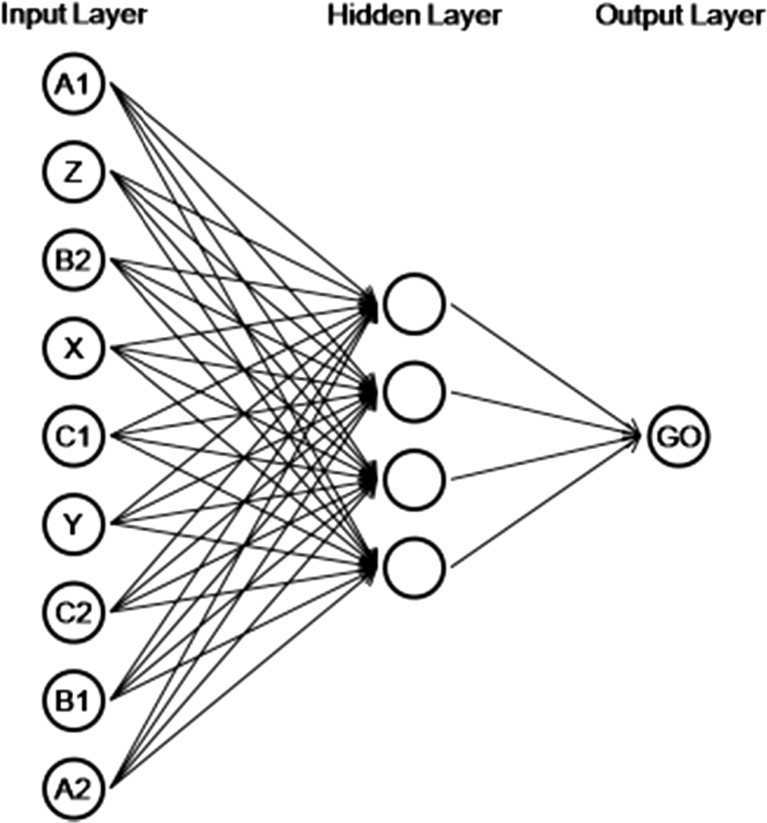
Network architecture of the computational model

The programming of the artificial neural network was performed utilizing the C Programming Language (Ritchie, Kernighan, & Lesk, 1975) and the Fast Artificial Neural Network Library (Nissen, 2003). Figure 1 presents a scheme of the neural network architecture developed. As Tovar and Torres (2012) had done, the units of the input layer were disposed to avoid two stimuli of the same class being adjacent.

Each unit of the input layer corresponded to a specific experimental stimulus. The activation of a unit with value 1 represented the presence of its corresponding stimulus in the trial, while the activation with value 0 represented its absence. The unit in the output layer made the representation of the emission of the *go* or *no-go* responses. During training, the output vector 1 was considered as the representation of the *go* response, whereas the output vector 0 was considered as the representation of the *no-go* response.

**Fig. 2:**
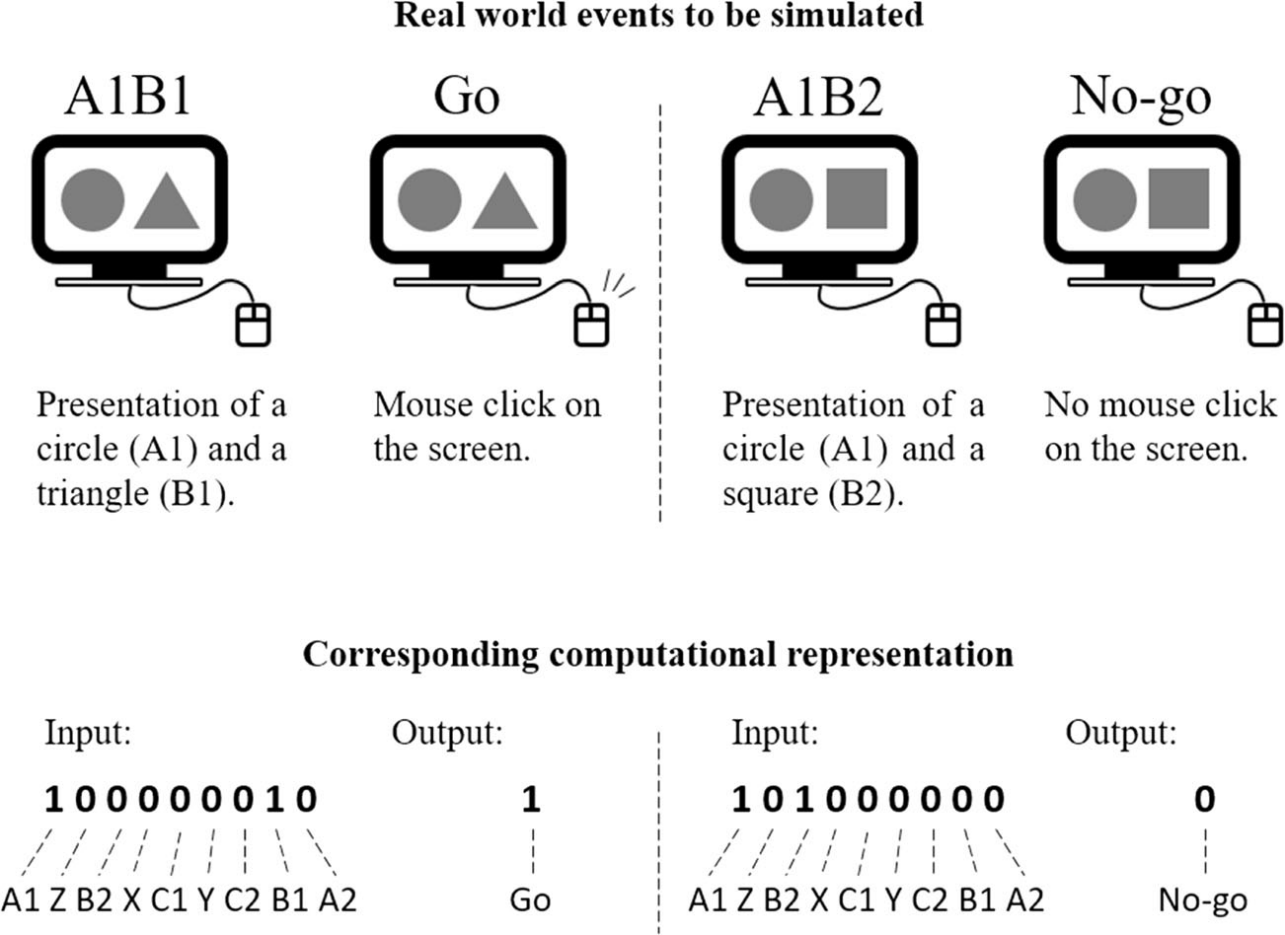
Illustration of two trials regarding stimuli presentation and response emission. On the upper part are the real-world events related to each trial. On the bottom part are the corresponding computational representations for each simulated event. *Left*: Presentation of the stimuli A1B1 and the emission of the *go* response. *Right*: Presentation of the stimuli A1B2 and the emission of the *no-go* response

Figure 2 illustrates the computational representation of two trials regarding stimuli presentation and response emission. To simulate the presentation of A1B1, the input vector activated the units that referred to A1 and B1 with value 1 and all the others with value 0. In the input layer, the first and eighth units corresponded to A1 and B1, respectively, so the input vector that represented A1B1 presentation was 100000010. Next, the unit in the output layer should ideally print the output vector 1, representing that the *go* response was emitted. In the same way, A1B2 presentation was represented by the input vector 101000000, since the first and third units of the input layer corresponded to A1 and B2, respectively. Then, the printed output vector would ideally be 0, representing that the *no-go* response was emitted.

An input vector became an output vector by a series of mathematical computations (see Haykin, 2009). The value of each unit in the input layer was passed to the units in the hidden layer, multiplied by the connection weight between them. Each connection between two units (lines in Fig. 1) had a different weight. The resulting values in each unit of the hidden layer were then adjusted to be in the interval between 0 and 1 by an activation function called *sigmoid*. This process was repeated to pass the values from the hidden to the output layer, generating an output vector.

**Fig. 3:**
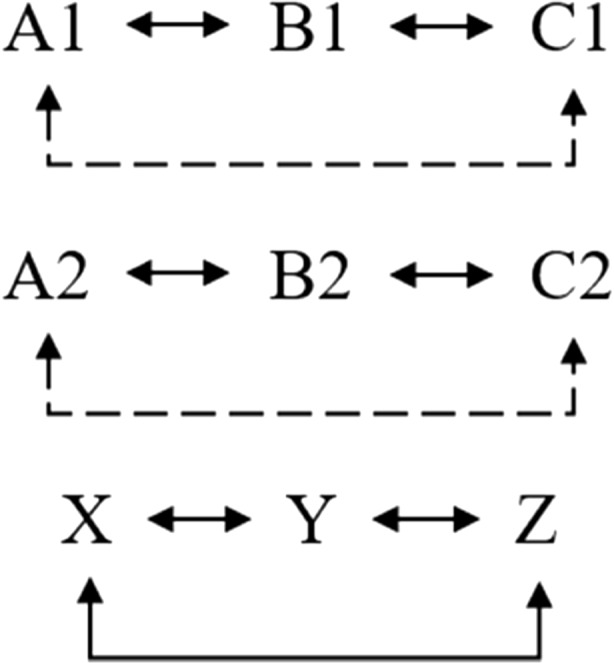
Trained relations (*solid line*) and tested relations (*dashed line*)

Two sets of stimuli were utilized for training and testing conditions. The additional class training utilized three stimuli (X, Y and Z) as in Tovar and Torres (2012). This training would correspond to the pre-experimental history simulation of a human participant with linguistic capacities (Lyddy & Barnes-Holmes, 2007). The relations XY, YZ and XZ were established. The transitivity relation (XZ) was directly trained to ensure establishment of the equivalence class XYZ. Using the second set of stimuli, the relations A1B1, A2B2, B1C1 and B2C2 were trained for the *go* response, as well as the relations A1B2, A2B1, B1C2 and B2C1 for the *no-go* response, as shown in Fig. 3.

The input vectors and their corresponding output vectors considered correct during the training phase, or expected during the testing phase, are presented in Table 1. Each input vector generated an output vector. During training, when the generated output vector did not correspond to the value considered correct in the trial (right side of Table 1), the connection weights between units were altered to produce output vectors closer to those desired on following trials. Thus, the connection weights, to which random values between 0 and 1 were initially attributed, would assume optimized values that progressively produced output vectors closer to those desired. The changes in the connection weights were made by the backward propagation algorithm (Rumelhart, Hinton, & Williams, 1986), with a learning rate of 0.3 and zero learning momentum—parameters that determine the magnitude of the change in the connection weights (see Haykin, 2009). The backward propagation consisted of a computational procedure analogous to the learning process involved in experiments with humans. The training phase ended when the stopping criterion RMS ≤ 0.0025 (root mean square) was met, meaning that all input vectors of the training phase were generating output vectors that were sufficiently close to the correct ones, with minimum error.

**Table 1:** Input vectors and their corresponding output vectors considered correct during the training phase or expected during the testing phase

Phase	Compound	Input vectors									Output vectors
		A1	Z	B2	X	C1	Y	C2	B1	A2	Go/no-go
Training	XY	0	0	0	1	0	1	0	0	0	1
Training	YZ	0	1	0	0	0	1	0	0	0	1
Training	XZ	0	1	0	1	0	0	0	0	0	1
Training	A1B1	1	0	0	0	0	0	0	1	0	1
Training	A1B2	1	0	1	0	0	0	0	0	0	0
Training	B1C1	0	0	0	0	1	0	0	1	0	1
Training	B1C2	0	0	0	0	0	0	1	1	0	0
Training	A2B2	0	0	1	0	0	0	0	0	1	1
Training	A2B1	0	0	0	0	0	0	0	1	1	0
Training	B2C2	0	0	1	0	0	0	1	0	0	1
Training	B2C1	0	0	1	0	1	0	0	0	0	0
Test	A1C1	1	0	0	0	1	0	0	0	0	0.85 ~ 1.00
Test	A1C2	1	0	0	0	0	0	1	0	0	0.00 ~ 0.15
Test	A2C2	0	0	0	0	0	0	1	0	1	0.85 ~ 1.00
Test	A2C1	0	0	0	0	1	0	0	0	1	0.00 ~ 0.15

In the testing phase, the backward propagation algorithm was deactivated, maintaining the connection weights constant. At this point, the emergence of the relations A1C1 and A2C2 for the *go* response was evaluated, and of the relations A1C2 and A2C1 for the *no-go* response. Adapting the criterion utilized by Tovar and Torres (2012), an error of 0.15 was tolerated from the ideally expected output vector. Values between 0.85 and 1 were considered as the representation of the *go* response. Inversely, values between 0 and 0.15 were considered as the representation of the *no-go* response. Intermediate values, between 0.15 and 0.85, were considered as absence of consistency in the emission of one of the responses in particular. Symmetry tests were not conducted because there was no representation for the spatial location of stimuli, as done by Tovar and Torres (2012). Thus, a relation (e.g., A1C1) and its symmetric relation (e.g., C1A1) had the same input vectors.

The training and testing steps were repeated in six runs, which, for the purpose of simulation, would be equivalent to six human participants.

### Results

**Fig. 4:**
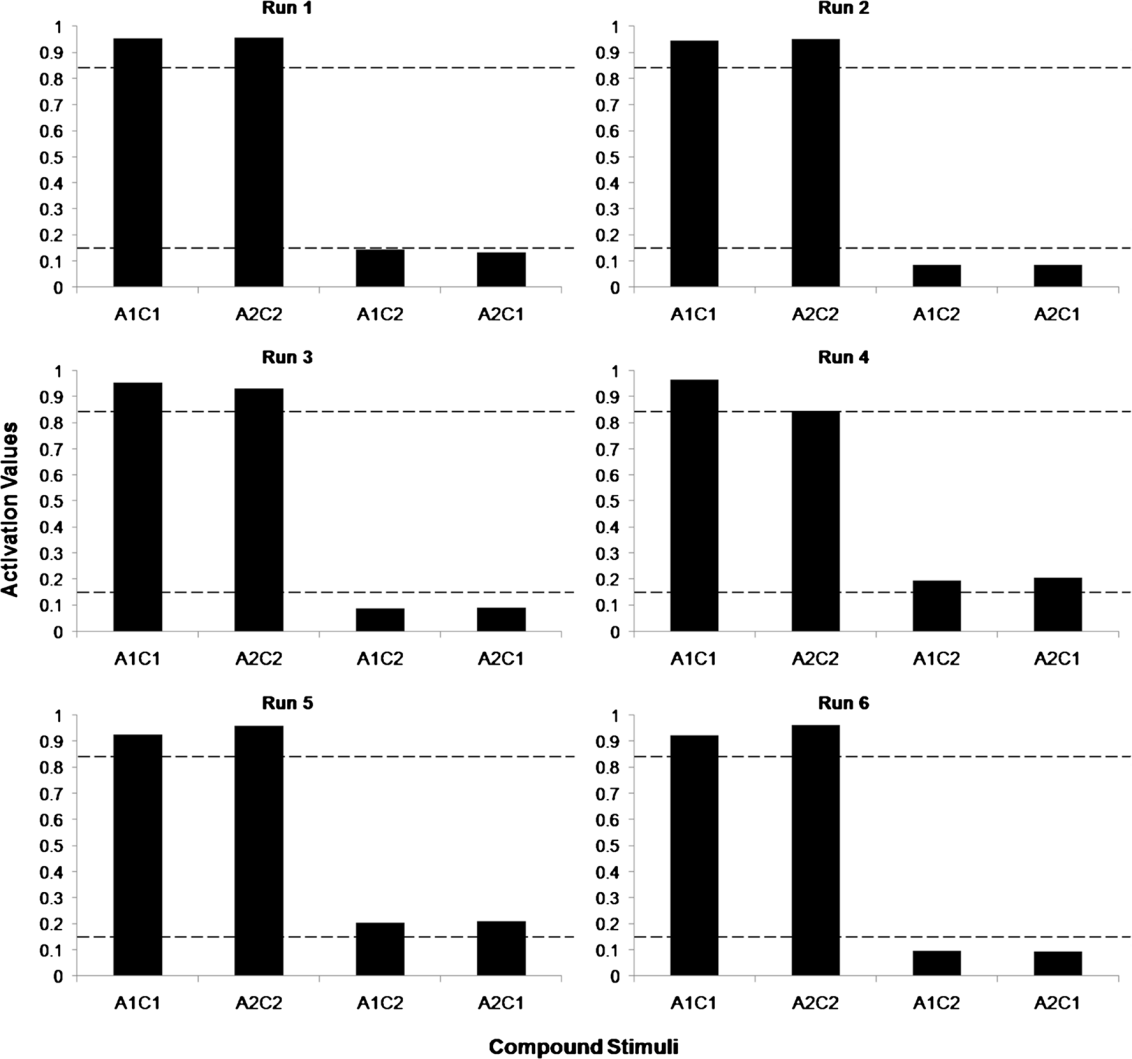
Activation values for each compound presented in the testing phase, in each run. The *upper dashed line* is the minimum threshold for the representation of the *go* response, and the *lower dashed line* is the maximum threshold for the representation of the *no-go* response

The training criterion was met after 526 epochs on average. Considering that 11 relations were trained in each epoch, the criterion for changing phase was met, on average, after 5,781 iterations. Figure 4 shows the activation values for each compound presented in the testing phase, in each run. The *go* response corresponds to values equal to or greater than 0.85 (upper dashed line), while the *no-go* response corresponds to values equal to or less than 0.15 (lower dashed line).

Four out of six runs met the criteria for the emergence of the AC relation, indicating the formation of the equivalence classes A1B1C1 and A2B2C2. The forth run satisfied neither the criterion for the *go* response for A2C2 nor that of the *no-go* response for A1C2 and A2C1. The fifth run met the criterion for the *go* response for A1C1 and A2C2, but did not meet that of the *no-go* response for A1C2 and A2C1.

### Discussion

Experiment 1 evaluated the possibility of utilizing artificial neural networks to simulate equivalence class formation using the go/no-go procedure with compound stimuli. It was found that four out of six runs satisfied the criteria for equivalence class formation. The result that not all runs met the criteria resembles the experiments in which the go/no-go procedure is utilized with humans, given that not all participants demonstrate equivalence class formation (e.g., Debert, Matos, & McIlvane, 2007; Perez, Campos, & Debert, 2009; Debert, Huziwara, Faggiani, De Mathis, & Mcilvane, 2009; Campos, Debert, Barros, & McIlvane, 2011; Grisante et al., 2013).

The results obtained were similar to those found in Tovar and Torres (2012), which simulated equivalence class formation utilizing the yes-no procedure, which also did not differentiate sample and comparison stimuli functions. Thus, the results of the current work aggregate to those of Tovar and Torres (2012) as evidence that it is possible to develop computational models to simulate equivalence class formation without the need to attribute specific functions to the antecedent stimuli.

All runs of Experiment 1 underwent the additional class training, as commonly takes place in experiments involving simulations of equivalence class formation (e.g., Barnes, & Hampson, 1993; Cullinan, Barnes, Hampson, & Lyddy, 1994; Lyddy & Barnes-Holmes, 2007; Lyddy, Barnes-Holmes, & Hampson, 2001; Tovar & Torres, 2012). It has been reported that the additional class training is indispensable for simulations using MTS (Barnes & Hampson, 1993) and yes-no (Tovar & Torres, 2012) procedures. However, it is still not known if the additional class training would also be a necessary step for simulations using the go/no-go procedure with compound stimuli. It would be important to evaluate whether, in the absence of this training, the results of the simulations utilizing the model developed in the present study would remain the same, or if, in the same manner as with other procedures, the additional class training would prove essential to the simulation of equivalence class formation.

## Experiment 2

Studies that simulated equivalence class formation using MTS and yes-no procedures noted that the additional class training is an indispensable step (e.g., Barnes & Hampson, 1993; Tovar & Torres, 2012). It is discussed that the additional class training is an analog to the pre-experimental experience that human participants have (e.g., Lyddy & Barnes-Holmes, 2007; Tovar & Torres, 2012). It is necessary to evaluate whether this training is also important when the simulations are made using the go/no-go procedure with compound stimuli.

Experiment 2 aimed to evaluate the possibility of simulating equivalence class formation using the go/no-go procedure with compound stimuli without performing the additional class training.

### Method

**Fig. 5:**
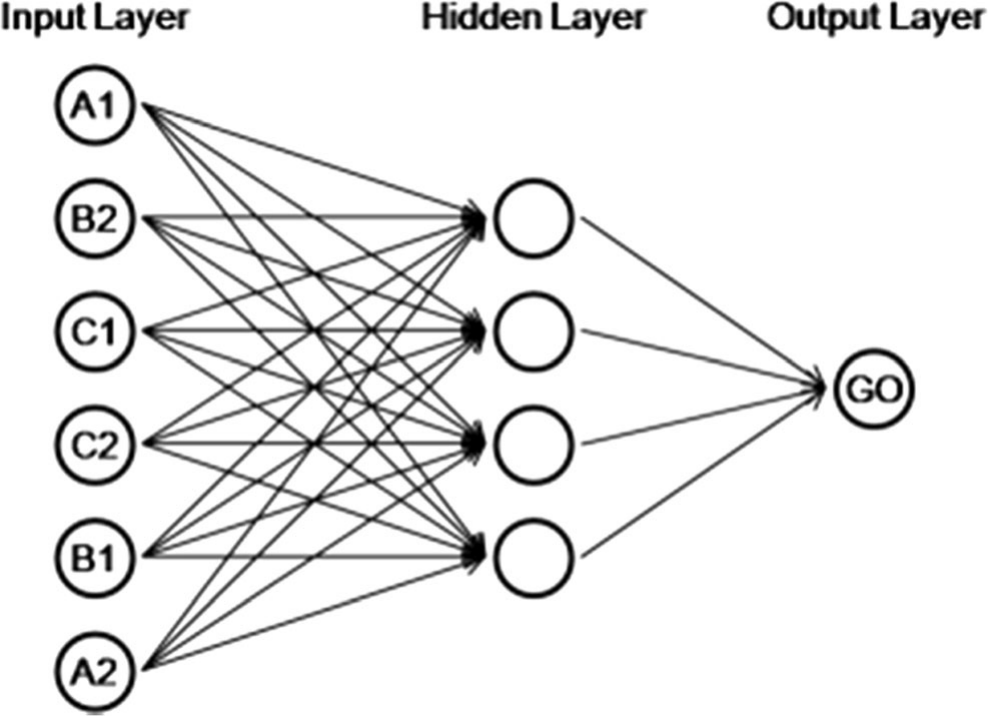
Network architecture of the model that did not undergo additional class training

The same training and testing procedures as in Experiment 1 were performed, except that there was no additional class training. The network architecture utilized is represented in Fig. 5. It was altered in order not to contain units referring to the stimuli X, Y and Z.

**Fig. 6:**
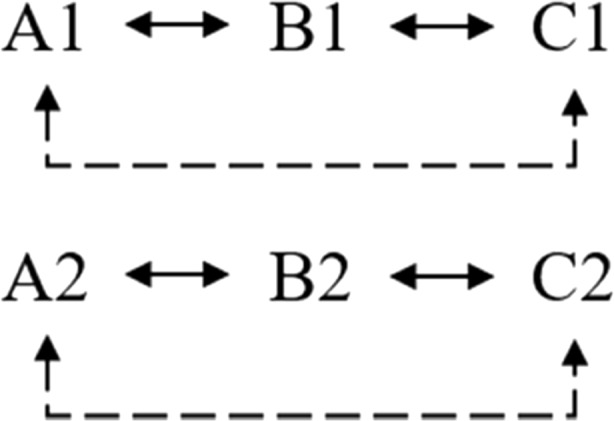
Trained relations (*solid line*) and tested relations (*dashed line*) of the simulations that did not undergo additional class training

The trained and tested relations are presented in Fig. 6.

The input vectors and their corresponding output vectors considered correct during the training phase, or expected during the testing phase, are represented in Table 2. The vectors were the same as those in Experiment 1, differing only in that they were neither representations for the stimuli X, Y and Z nor steps related to the additional class training.

**Table 2:** Input vectors and their corresponding output vectors considered correct during the training phase, or expected during the testing phase, for the simulation that did not involve additional class training

Phase	Compound	Input vectors						Output vectors
		A1	B2	C1	C2	B1	A2	go/no-go
Training	A1B1	1	0	0	0	1	0	1
Training	A1B2	1	1	0	0	0	0	0
Training	B1C1	0	0	1	0	1	0	1
Training	B1C2	0	0	0	1	1	0	0
Training	A2B2	0	1	0	0	0	1	1
Training	A2B1	0	0	0	0	1	1	0
Training	B2C2	0	1	0	1	0	0	1
Training	B2C1	0	1	1	0	0	0	0
Test	A1C1	1	0	1	0	0	0	0.85 ~ 1.00
Test	A1C2	1	0	0	1	0	0	0.00 ~ 0.15
Test	A2C2	0	0	0	1	0	1	0.85 ~ 1.00
Test	A2C1	0	0	1	0	0	1	0.00 ~ 0.15

### Results

**Fig. 7:**
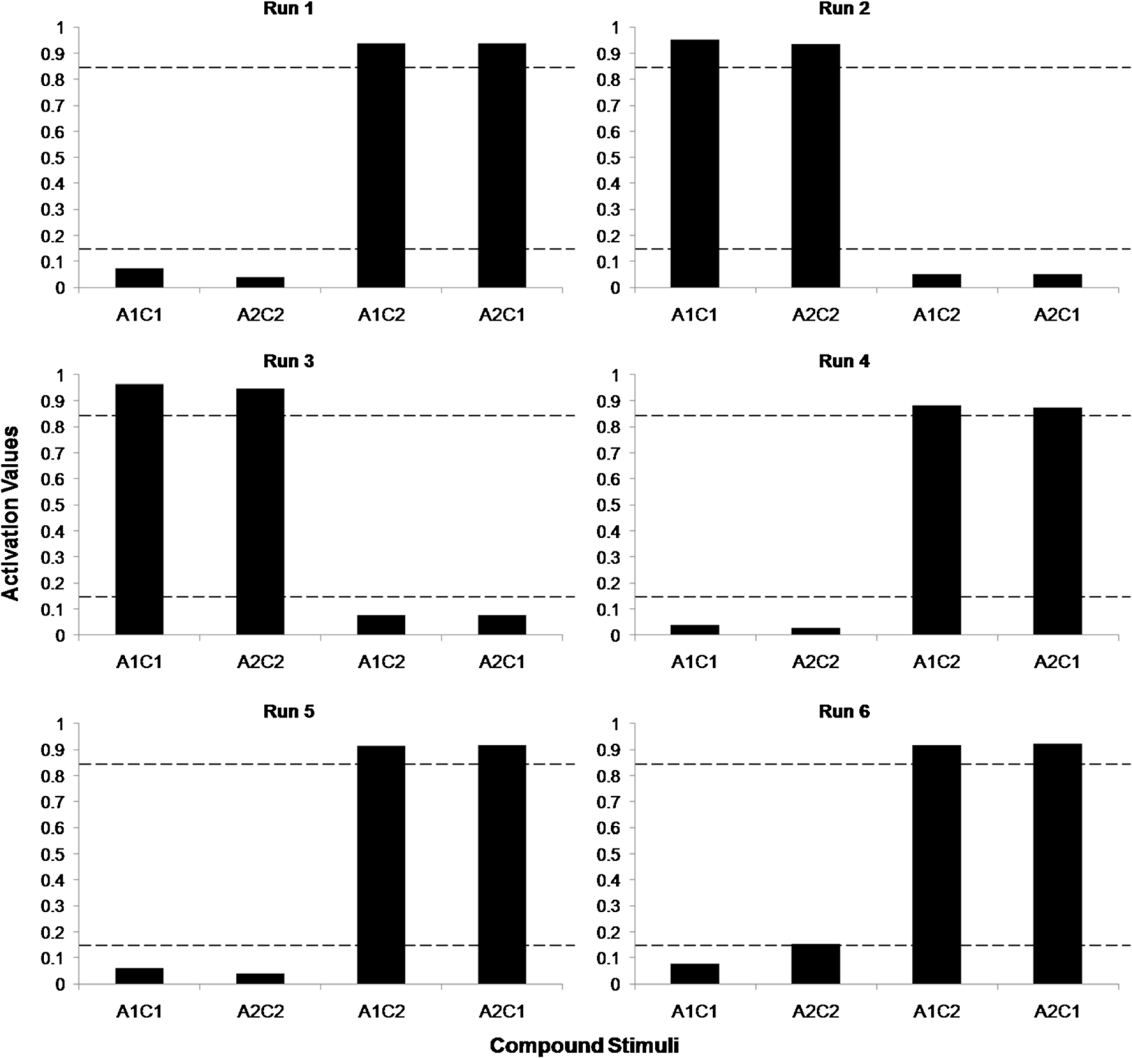
Activation values for each compound presented in the testing phase, in each run. There was no additional class training. The *upper dashed line* is the minimum threshold for the representation of the *go* response, and the *lower dashed line* is the maximum threshold for the representation of the *no-go* response

Training criterion was met, on average, after 8737 iterations (or 1092 epochs). Figure 7 shows the activation values for each compound presented in the testing phase in each run.

Only two out of six runs satisfied the criteria for the emergence of the AC relation, indicating the formation of the equivalence classes A1B1C1 and A2B2C2. It is noteworthy that the second, fourth and fifth runs fulfilled the criterion for the *go* response for compounds containing elements of different classes (A1C2 and A2C1), whereas they satisfied the criterion for the *no-go* response for compounds containing elements of the same class (A1C1 and A2C2). This result was the inverse of what was expected in the transitivity test. In the sixth run, if the criterion for the *no-go* response for A2C2 had been met, this execution would also have presented an inverse pattern to what was expected in the testing phase.

### Discussion

Experiment 2 evaluated the effect of the absence of the additional class training on the results of the equivalence class formation simulation using the go/no-go procedure with compound stimuli. In the training phase, the criterion for phase change was met, on average, after 8,737 iterations, a number significantly greater in comparison with the average of 5,781 iterations required in Experiment 1. The increase in iterations to fulfill the criterion in Experiment 2 indicates that the additional class training facilitates the establishment of directly trained relations. In the testing phase, it was found that only two out of six runs satisfied the criteria for equivalence class formation. Besides this, three of the runs that did not demonstrate equivalence class formation presented a response pattern that was inverse to what was expected in the testing phase. In other words, the criterion for the *go* response was met for compounds containing elements from different classes, whereas the criterion for the *no-go* response was satisfied for compounds containing elements from the same class. These results are very different from those observed in experiments with humans using the go/no-go procedure with compound stimuli (e.g., Debert, Matos, & McIlvane, 2007; Perez, Campos & Debert, 2009; Debert, Huziwara, Faggiani, De Mathis, & Mcilvane, 2009; Campos, Debert, Barros, & McIlvane, 2011; Grisante et al., 2013). Therefore, the additional class training is essential to simulate equivalence class formation when using the go/no-go procedure with compound stimuli in the same way that it is also necessary for MTS (e.g., Barnes & Hampson, 1993) and yes-no (Tovar & Torres, 2012) procedures.

Tovar and Torres (2012) also found that, when the additional class training did not occur, some runs presented a response pattern inverse to what was expected in the testing phase. The authors raised the hypothesis that the inverse pattern appeared because, in transitivity tests, the probability of the occurrence of the *yes* response was the same as that of the *no* response, when the additional class training was not performed. This is because, as pointed out by Fields et al. (2009), in the case of humans, baseline training with pairs of stimuli A1B1 and B1C1, associated with the *yes* response, establishes the condition for occurrence of the *yes* response in the presence of A1C1. However, baseline training with the pairs A1B2 and B2C1 also establishes the condition for the *no* response to occur in the presence of A1C1. Thus, it is possible to explain inverse pattern emergence when there is no additional class training. Future studies could investigate this hypothesis.

## General Discussion

This work evaluated the possibility of utilizing artificial neural networks to simulate equivalence class formation using the go/no-go procedure with compound stimuli. In Experiments 1 and 2, it was found that it is possible to simulate equivalence class formation using the go/no-go procedure with compound stimuli. Also, it was observed that the additional class training is a necessary step of the simulation and likewise happens when other procedures are used (e.g., Barnes & Hampson, 1993; Cullinan, Barnes, Hampson, & Lyddy, 1994; Lyddy & Barnes-Holmes, 2007; Lyddy, Barnes-Holmes, & Hampson, 2001, Tovar & Torres, 2012). As the model developed in the present study does not differentiate sample and comparison stimuli, it was not necessary to utilize a computational resource similar to the sample-marking duplicator used in the networks derived from RELNET (e.g., Barnes & Hampson, 1993). Since the output vectors were generated only by the information about the stimuli presented, the results obtained in the testing phase represented emergent responses. Therefore, it can be affirmed that the model developed is an alternative to RELNET for the study of equivalence relations using computational simulations.

The present study provided the methodological novelty of utilizing a single unit in the output layer. In the yes-no procedure, the two possible responses are independent and therefore each one needs an independent computational representation. It is required to make the representation of two distinct responses, a *yes* response and a *no* response. For this reason, Tovar and Torres (2012) developed a network architecture in which the output layer had two units. The computational representation of the *yes* response was made by the output vector (1, 0), that is, the activation of the *yes* unit with value 1 and the activation of the *no* unit with value 0. The computational representation of the *no* response was made in reverse by the output vector (0, 1), that is, the activation of the *yes* unit with value 0 and the activation of the *no* unit with value 1. Although this network architecture is conceptually correct, given the independence between the *yes* and *no* responses, computationally there is redundancy: whenever the *yes* unit is activated with value 1, it is expected that the *no* unit is necessarily activated with value 0, and, inversely, whenever the *yes* unit is activated with value 0, it is expected that the *no* unit is activated with value 1. Therefore, the existence of two units in the output layer is computationally redundant. It would be more parsimonious and would simplify the computational training procedure if, in the output layer, there were only one unit. This was possible when developing a network architecture utilizing the go/no-go procedure with compound stimuli. In this procedure, the two possible responses, *go* and *no-go*, are dependent on each other, given that, conceptually, in a certain trial the *no-go* response cannot be emitted if the *go* response was emitted, and vice-versa. Due to this, it was conceptually correct to propose a network architecture containing only one unit in the output layer. The activation of the only unit in the output layer with value 1 was the computational representation of the *go* response, and the activation of this unit with value 0 was the computational representation of the emission of any response different from *go*, in other words, the *no-go* response. This network architecture containing only one activation unit in the output layer avoids computational redundancies and is more parsimonious. Although in simple network architectures this methodological novelty may not impact performance, it could make a difference in simulations using more complex architectures or weak hardware.

During the testing phase, the backward propagation algorithm was deactivated, as commonly occurs in simulations of equivalence class formation (e.g., Barnes & Hampson, 1993; Cullinan, Barnes, Hampson, & Lyddy, 1994; Lyddy & Barnes-Holmes, 2007; Lyddy, Barnes-Holmes, & Hampson, 2001; Okada, Sakagami, & Yamakawa, 2005; Tovar & Torres, 2012). However, in this mode of simulating the testing phase, the connection weights remain constant during all testing trials, preventing variations in the output vectors that are generated. The results remain the same, irrespective of test characteristics (for example, the order of the relations tested or the quantity of re-tests conducted). Thus, an important limitation of the present model is that it is neither capable of reproducing results obtained in experiments involving humans, in which it was found that the order of the relations tested affects the emergence of equivalence classes (e.g., Adams, Fields, & Verhave, 1993), nor results in which the emergence of equivalence classes occurs when the testing phase is repeated without additional training steps (e.g., Debert, Matos, & McIlvane, 2007; Perez, Campos, & Debert, 2009). Another limitation of the present model is the absence of spatial representation for the stimuli presented, just as in Tovar and Torres (2012). Without representing the spatial location, it is not possible to conduct symmetry tests, as the input vector of a relation and its symmetric relation are the same. However, such tests are conducted in the majority of studies with humans using the go/no-go procedure with compound stimuli (e.g., Debert, Matos, & McIlvane, 2007; Perez, Campos, & Debert, 2009; Debert, Huziwara, Faggiani, De Mathis, & Mcilvane, 2009; Campos, Debert, Barros, & McIlvane, 2011; Grisante et al., 2013).

It is suggested that future studies could evaluate the possibility of simulating the training and testing trials of conditional relations considering the spatial location of the stimuli, but seeking to avoid computational resources as the sample-marking duplicator, characteristic of the networks derived from RELNET. Representation of the stimuli spatial location would make the simulation more similar to what has been done with humans using the go/no-go procedure. This would enable, for example, conducting symmetry tests (Debert, Matos, & McIlvane, 2007; Perez, Campos, & Debert, 2009; Debert, Huziwara, Faggiani, De Mathis, & Mcilvane, 2009; Campos, Debert, Barros, & McIlvane, 2011; Grisante et al., 2013). It is also suggested that future studies could investigate the possibility of utilizing some algorithm or other computational resource that promotes variability of the output vectors during the testing phase. This could allow simulating, for example, the effect of the order in which relations are tested, or the effect of re-testing, on the probability of equivalence class formation. Experiments with humans revealed that, in the go/no-go procedure with compound stimuli, the training structure has no effect on the equivalence class emergence (Grisante et al., 2013), unlike what happens in the MTS procedure (e.g., Adams, Fields, & Verhave, 1993). It is further suggested that there could be future investigations regarding whether the model developed in the present study is capable of reproducing these results obtained with humans using the go/no-go procedure with compound stimuli, comparing it with the study of Lyddy and Barnes-Holmes (2007), which simulated the effects of the training structure on the emergence of equivalence classes using MTS. The present study used the RMS as a stopping criterion during the training phase, as it is commonly performed when simulating equivalence class formation (e.g., Barnes & Hampson, 1993; Cullinan, Barnes, Hampson, & Lyddy, 1994; Lyddy & Barnes-Holmes, 2007; Lyddy, Barnes-Holmes, & Hampson, 2001; Tovar & Torres, 2012). It is worth mentioning that one alternative to the RMS is a cross-validation test, such as the k-fold cross-validation (see Mitchell, 1997). Future studies could investigate whether an alternative stopping criterion would bring any advantage to the field. Furthermore, it could be evaluated whether it is possible to simulate the formation of larger classes. The establishment of classes containing more than three stimuli would make it viable to investigate new aspects of the phenomenon studied, such as using simulations to investigate specificities of the nodal distance effect. Finally, future studies could evaluate the possibility of adapting the present model for the simulation of behaviors involving contextual control. So far, no alternatives to RELNET (Barnes & Hampson, 1993) exist for the simulation of such behavior.
